# Insulin Modifies Honeybee Worker Behavior

**DOI:** 10.3390/insects3041084

**Published:** 2012-10-24

**Authors:** Christine M. Mott, Michael D. Breed

**Affiliations:** Department of Ecology and Evolutionary Biology, The University of Colorado, Boulder, CO 80309, USA; E-Mail: c_mott44@yahoo.com

**Keywords:** insulin, *Apis mellifera*, honeybee, division of labor, sucrose response threshold, foraging

## Abstract

The insulin signaling pathway has been hypothesized to play a key role in regulation of worker social insect behavior. We tested whether insulin treatment has direct effects on worker honeybee behavior in two contexts, sucrose response thresholds in winter bees and the progression to foraging by summer nurse bees. Treatment of winter worker bees with bovine insulin, used as a proxy for honeybee insulin, increased the bees’ sucrose response threshold. Treatment of summer nurse bees with bovine insulin significantly decreased the age at which foraging was initiated. This work provides further insight into the role of endocrine controls in behavior of in honeybees and insects in general.

## 1. Introduction

Endocrine function is currently a topic of great interest in the study of social insects. Hunt *et al.* [1], Hunt and Amdam [2], and Amdam *et al.* [3] proposed the Reproductive Ground Plan Hypothesis (RGPH), which attempts a comprehensive explanation for the linkages between worker reproductive physiology and behavior. The RGPH suggests that regulatory pathways, including the insulin/insulin-like signaling system (IIS), that regulate reproduction in solitary insects also contribute to age-related changes in worker task performance in social insects [4].

In social insects nutrition, age, and reproductive status interact to regulate worker behavior [3]. Temporal polyethism (age-related division of labor) in honeybees results in young adult workers performing tasks in the nest such as nursing, while middle-aged bees perform tasks on the periphery of the nest which include of building comb, processing food, guarding or fanning. The oldest worker bees are foragers [5,6,7,8,9]. Changes in nutritional status, which are regulated through the insulin signaling system, play key roles in dictating behavioral shifts as workers age, probably interacting with social cues [10,11].

The yolk protein, or vitellogenin, system is thought to contribute to regulation of both reproduction and behavior in honeybee workers [3]. The IIS may have an additional role in behavioral regulation, as vitellogenin production correlates inversely with insulin production, and artificial suppression of vitellogenin accelerates behavioral progression in workers [12]. Older worker bees, such as foragers, have high insulin and low vitellogenin production while younger bees, such as nurses, have low insulin and high vitellogenin production [3,13,14,15].

Insulin production may also provide one of the links, along with other regulatory systems such as juvenile hormone, between individual worker nutritional status and behavioral task group [10,11,16,17]. For example, Toth and Robinson [11] and Schulz *et al.* [17] hypothesized that older bees progress to foraging because lipid stores decrease with age, which correlates with increasing insulin production. Amdam *et al.* [5] demonstrated that nursing behavior accelerates foraging onset by approximately 1 to 1.5 days in high brood *versus* no brood honeybee colonies. Wang *et al.* [12] noted that bees with supernumary ovaries terminated brood care earlier than controls, but did not show a difference in vitellogenin transcript levels. This may be indicative of some other pathway affecting behavioral ontongeny from solely a nutritional perspective. Demonstrated changes in nutrition or other effects of changing additional hormonal pathways, such as IIS, may cause this acceleration of age‑related task performance. Worker nutritional status can be measured using an assay, sucrose response threshold (SRT), associated with feeding behavior. This commonly used bioassay tests worker responsiveness to sucrose concentration [8,18], and may be indicative of changing nutritional status among honeybee workers. High sucrose response thresholds (low responsiveness, 0.9 M to 1.5 M sucrose) are often found in foragers, known to have high insulin production and low lipid stores, and low sucrose response thresholds (high responsiveness, 0.003 M to 0.03 M sucrose) are found in young and middle‑aged bees [18] and may correlate with the lower insulin production and greater lipid stores.

Age- and task-related changes in social insect worker behavior and physiology are generally irreversible [6]. However, older worker honeybees in colonies faced with a severe shortage of nurse bees can revert to that task, though the reversion may be incomplete or for a limited length of time [19]. Consistent with this, winter bees are long-lived workers who form the core of the work force for the colony in the spring when brood rearing resumes. Older winter bees can adopt young bee tasks, such as brood care, to restart colony production at the beginning of the spring transition period to brood-rearing and summer foraging [20]. The mechanism for this behavioral transition is unknown, but IIS may be one underlying mechanism controlling physiology and behavior in honeybees.

We tested whether manipulation of insulin titers in honeybees (*Apis mellifera*) affects behavior related to nutrition and task performance. We measured the effect of insulin treatment in expression of worker behavior in two contexts. The first was sucrose responsiveness of winter and summer worker bees, and the second was the timing of foraging initiation in summer worker bees. Our three hypotheses for this study are: Winter bees will show an increased SRT following insulin treatment, summer season middle-aged guards and old-aged pollen foragers will respond to insulin treatment with less dramatic changes in SRT, and summer nurse bees treated with insulin will more quickly progress to foraging than untreated controls.

## 2. Results and Discussion

### 2.1. Winter Bees

**Figure 1:**
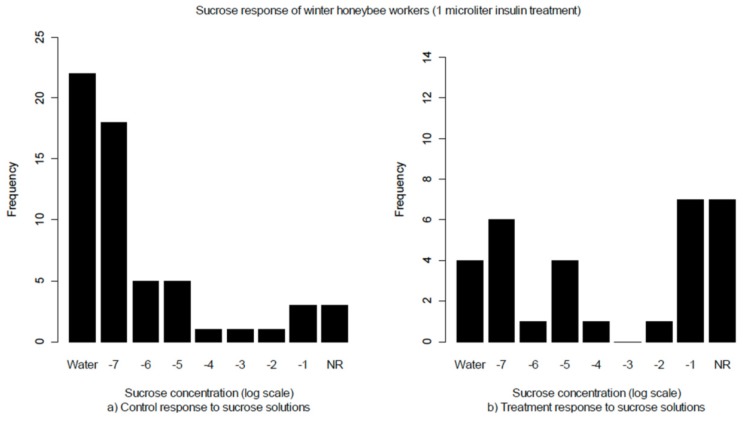
Sucrose response threshold of winter honeybee workers. The two types of controls, untreated and sham treated, did not differ significantly and are grouped together in the figure. NR = no response. There is a significant difference between the sucrose response thresholds of the controls (**a**, n = 60) and insulin treatments (**b**, n = 31) (X^2 ^= 6.4532, *p* = 0.040).

We predicted that altering the balance of insulin in the haemolymph in winter workers would modify feeding behavior. In response to insulin treatments we found a behavioral change indicated by a shift in SRT from most of the bees responding to water or very low sucrose concentrations in the controls to bees either responding to high sucrose concentrations or not responding at all in the treatments (Figure 1). A Chi-square test indicated that the proportion of bees responding in the assay is not independent of treatment group, indicating a significant effect of the insulin treatment. 

### 2.2. Summer Bees

We predicted that because older bees already have high levels of insulin they would be less sensitive to direct insulin treatments and would be less likely to express changes in task-related feeding responses [11,12,13]. Chi-square tests comparing the sucrose response thresholds of the 1- and 2‑microliter injected guard groups to controls showed no significant effect (*p* = 0.2983, *p* = 0.2726). This was also true for foragers (*p* = 0.2983). These results show no effect of insulin treatment on sucrose response threshold for either the guard or forager task groups.

### 2.3. Age-Related Changes in Task

**Figure 2:**
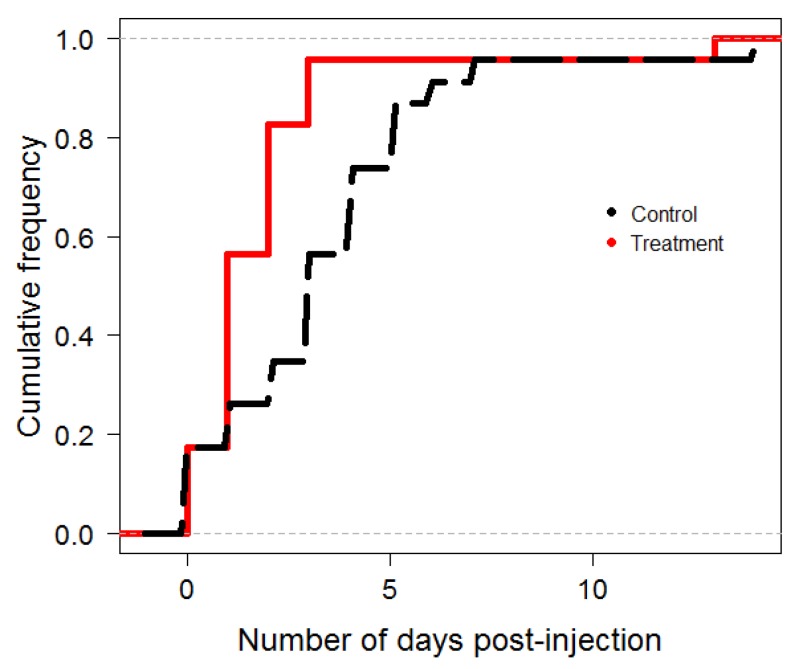
Cumulative fraction plot for Kolmogorov-Smirnov comparison of nurse bees treated with 1 microliter of insulin (solid line) and sham control nurse bees (dashed line). The Y-axis is the cumulative frequency of bees that foraged on a given day post-injection. The plot shows a significant difference in number of days to foraging, as measured by extended flight activity, between treatment with one microliter of insulin (solid line, n = 23) and sham-treatment with 1 microliter of buffer solution (dotted black line, n = 23) nurse bees (KS Test values D = 0.4783, *p*-value = 0.010, bootstrap *p*‑value = 0.004). The mean time to forage among untreated bees was 3.4 days, and the mean time to forage for treated bees was 1.6 days post-injection, indicating that insulin accelerated foraging behavior in treated bees by nearly two days.

We next tested the effects of insulin on age-related task performance. Bees which no longer have enough vitellogenin to rear brood are expected to transition through middle-aged tasks and turn finally to foraging, a transition which may be linked to insulin activity. Figure 2 shows the significant decrease between post-injection time to foraging between control (n = 23) and treatment (n = 23) groups. A Kolmogorov-Smirnov test indicated that the distribution of the treatment data differed significantly from the control data in post-injection time to forage (D = 0.4783, *p* = 0.010).

Previous honeybee research has focused on behavioral manipulation at the genetic level, and many studies indicate that manipulation of the IIS pathways disrupts energy homeostasis and alters behavior. Wang *et al.* [21] demonstrated that food-related behavior was altered by an insulin receptor substrate (IRS) protein gene knockdown that altered IIS signals to peripheral tissues. Our results also provide insight into behavior alterations involving the IIS pathways, but through direct manipulation of insulin blood titers. As predicted, injected winter bees showed high responsiveness to the hormone through altered sucrose response. Specifically, winter bees in the control groups show a distinct preference for water or very low concentration of sucrose, while the injected individuals responded only to much higher sucrose concentrations. This change may be due to low initial insulin production in our experimental subjects. If insulin production changes in workers through their lifecycle, similar to the changes predicted by the RGPH for vitellogenin, then vitellogenin production and associated behaviors may also be affected by insulin. Speculatively, low insulin levels predicted in winter workers may allow reversion to young-bee nursing behavior in the spring. Amdam *et al.* [22] suggested that the significant buildup of vitellogenin in tissues of European honeybee workers during brood-rearing and in winter is a survival mechanism and may increase brood-rearing success. They proposed that low insulin activity during winter maximizes vitellogenin storage, enabling brood care in the spring. Alternatively, insulin may directly affect blood trehalose (blood sugar) levels, which may increase their ability to retain any available water and survive in osmotically challenging conditions for long periods [20,23]. The intense and immediate response to water among the winter controls may be indicative of this osmotic stress. 

Sucrose response thresholds of summer bees showed little response to insulin treatment. The negative results in guards were surprising, as we expected middle-aged bees to also maintain moderate insulin titers. They may, however, be farther along in the age-related changes in the endocrine cycle than suggested by Corona *et al.* [13] and Hunt and Amdam [2]. These models suggested that bees of intermediate age would still have endocrine profiles matching those of younger bees, and that the endocrine transition to a forager-like profile would occur after activities like guarding. We found a significant acceleration of foraging onset in insulin-treated nurse bees, which progressed to foraging a mean of 1.56 days earlier than control bees. Schulz *et al.* [17] argued that young bees require some developmental period before they progress to foraging, but the fact that nearly 20% of our treated nurse bees foraged at zero days post-injection in this experiment suggests there may be other factors to consider than simply chronological age, to include hormonal changes. 

Our results suggest that there may be a link between precocious foraging and the effects of insulin. Insulin is upstream of both juvenile hormone and vitellogenin in the pathway modeled by Corona *et al.* [13], and may be driving the system for the behavioral effects of these other hormones. Our findings clearly establish a role for insulin in the regulation of honeybee worker task performance, and support the concept that nutritional status affects social insect worker behavior.

## 3. Experimental Section

**Figure 3:**
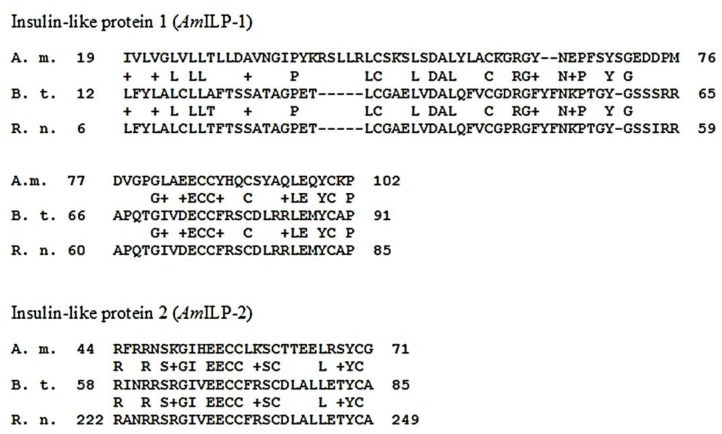
Protein alignments for *Apis mellifera (A.m.)*, *Bos taurus (B.t.)*, and *Rattus norvegicus (R.n.)*, demonstrating highly conserved sequence among insulin-like peptides among multiple taxa. Plus signs indicate areas of substitution to a functionally similar amino acid.

We used bovine insulin as a proxy for honeybee insulin, following other research on arthropods [24,25,26,27]. To test the acceptability of bovine insulin as a proxy, we obtained DNA sequences for two honeybee insulin-like peptides, *Am*ILP-1 and *Am*ILP-2, from BeeBase Assembly 4.0 (GB17332 and GB10174. We compared protein translations and alignments for *Am*ILP-1 and *Am*ILP-2 (accessions AAF72409, AAA41386; NP_776512, EDM12158) to insulin sequences for cattle and rats. One segment in each peptide, 83 and 27 amino acids in length respectively, aligned closely across insect species and with the selected vertebrate species with 50% identity and 60% positives in each case (Figure 3). These peptide segments are more conserved than we would anticipate from random changes over time, leading us to believe that it is a potential receptor binding site within the insulin peptides. In addition, the honeybee protein showed several amino acid substitutions, with most changes to amino acids of similar properties. 

We used three distinct task groups for the sucrose response experiments: Winter bees, guards, and foragers. We began with workers collected from eight honeybee colonies in winter 2009, with four workers per sample, and transporting each individual in a separate vial containing a water source. We randomly assigned each individual to one of three experimental groups: control, buffer (procedural control), or insulin (treatment). We injected workers in the buffer group with 1 microliter of HEPES buffer solution (Fluka) to control for handling stress and injection response. Individuals in the insulin group received a 1-microliter of insulin injection (Sigma, bovine, 10 mg/mL, 25 mM HEPES buffer). We injected all solutions directly into the hemocoel, and then tested each individual across successive sucrose solutions, 10^−7^ to 0.1 Molar, for 2 minutes per solution or until a proboscis extension reflex (PER) occurred (as in [8,18]). We used 1-microliter injections for all task groups and repeated the experiment on the guard group using a 2-microliter injection.

Sucrose response thresholds were determined using well-established methods [8,18]. We used a 15 cm by 30.5 cm clear acrylic sheet, with eight 0.9 cm diameter wells. One well contained water, and the seven remaining wells contained a series of 10^−7^ to 0.1 Molar sucrose solutions, placed in the wells in order of increasing concentration. Bees were placed under a 5 cm clear petri dish top (0.64 cm deep); within this space the bee could move freely and, when the dish was placed over a well, the bee had the opportunity to sample the solution. The bee was moved to each successive solution for 2 minutes (as tested in a pilot experiment), or until a proboscis extension reflex (PER) occurred (as described by Pacheco and Breed [8] and Scheiner *et al. *[18]). A PER was defined as a full extension of the proboscis while drinking from or around the wells. We recorded the concentration of the first solution (lowest concentration) to elicit the response. Data were compiled for each experimental group and analyzed using the R statistics package. We used a Pearson’s Chi-squared test to determine if there was a difference in sucrose response threshold response between the experimental groups. We found no significant difference in response between the control and procedural control (X‑squared = 10.5174, df = 8, *p*-value = 0.230), and combined the control results into a single group for further analyses. 

For the task performance experiment, we collected a total of 279 nurse bees from three adjacent colonies in August 2009. Nurse bees were identified as those individuals performing a task with their head in a brood cell, and we confirmed the presence of larvae in the cell upon collection of the worker. Previous work indicated no effect of the buffer solution on the control group outcomes, so individuals were assigned to a procedural control or treatment group. We numbered each worker (BetterBee Queen numbers) and injected the individual with 2 microliters of the appropriate solution, recording the date, amount injected, and individual tag number and color before release to the colony. We observed the colonies each day from 19-08-09 to 14-09-09, in random three-hour blocks between 0900 and 1800, recording each tagged bee’s identification, task, and date (n = 23 for each group, a 16.5% return rate). Tagged individuals observed departing the hive on multiple flights or returning with a pollen load were reported as foragers, while individuals observed performing aggressive guarding behavior at the exit of the hive were reported as guard bees. We used a two-tailed Kolmolgorov-Smirnov test of distributions to determine if the probability densities of the treatment data and control data were the same. This allows the program to compare non-normally distributed data that are not entirely continuous, and to eliminate the problem of ties between distributions by creating a continuous distribution based on the observed data.

## 4. Conclusions

Our two main conclusions from this study are: (1) Winter workers show significant changes in SRT in response to insulin treatment, and (2) Nurse bees become precocious foragers when treated with insulin. This response contrasted with the minimal shift in response to insulin treatment seen in middle- and old-aged summer bee SRTs. Nurse bees progressed to foraging in a mean time of 1.6 days, less than half the transition time of controls, indicating a substantial effect of insulin on the behavioral transitions in worker bees.
